# circFKBP8(5S,6)-encoded protein promotes stress susceptibility in mice by down-regulating dopamine D3 receptor expression and its downstream AMPK/mTOR/ULK1 autophagy signaling

**DOI:** 10.1016/j.gendis.2025.101718

**Published:** 2025-06-18

**Authors:** Dandan Xu, Zihan Huang, Gaojia Zhang, Jiao Jiao, Yujia Cao, Mengyu Liu, Yan Kong, Zhijun Zhang

**Affiliations:** a Department of Neurology in Affiliated Zhongda Hospital and Jiangsu Provincial Medical Key Discipline, School of Medicine, Research Institution of Neuropsychiatry, Key Laboratory of Developmental Genes and Human Disease, Southeast University, Nanjing, Jiangsu 210009, China; b Shenzhen Key Laboratory of Precision Diagnosis and Treatment of Depression, Department of Mental Health and Public Health, Faculty of Life and Health Sciences, Shenzhen University of Technology, Shenzhen Institute of Advanced Technology, Chinese Academy of Sciences, Shenzhen, Guangdong 518055, China; c Department of Biochemistry and Molecular Biology, School of Medicine, Southeast University, Nanjing, Jiangsu 210009, China; d Department of Psychology and Sleep Medicine, The Second Hospital of Anhui Medical University, Hefei, Anhui 230061, China

**Keywords:** Autophagy, circRNA-encoded protein, circRNAs, Dopamine D3 receptor, Major depressive disorder

## Abstract

Major depressive disorder (MDD) is a serious mental disorder, yet the mechanism by which circular RNAs (circRNAs) are involved in the pathogenesis of MDD by encoding proteins is unknown. Our previous study has shown that circFKBP8(5S,6) relies on its encoded protein, namely cFKBP8, to promote susceptibility to chronic unpredictable mild stress (CUMS) in mice, but the precise molecular mechanisms are unknown. Here we found that overexpression of circFKBP8(5S,6) or cFKBP8 in neurons of the prelimbic cortex (PrL) of CUMS mice down-regulated the expression levels of DRD3 and its downstream AMPK/ULK1 (Ser555) and AMPK/mTOR/ULK1 (Ser757) pathways, which resulted in down-regulation of neuronal autophagy levels. Interestingly, both the activation and overexpression of DRD3 ameliorated the exacerbation of depressive-like behaviors induced by circFKBP8(5S,6) or cFKBP8, activated both the AMPK/ULK1 (Ser555) pathway and the AMPK/mTOR/ULK1 (Ser757) pathway, and up-regulated neuronal autophagy levels. In conclusion, circFKBP8(5S,6) or cFKBP8 promotes susceptibility to CUMS in mice, at least in part, by down-regulating DRD3 expression and its downstream AMPK/mTOR/ULK1 signaling pathway-mediated neuronal autophagy.

## Introduction

Circular RNAs (circRNAs) are non-coding RNAs, covalently closed circular RNA molecules formed by back-splicing of precursor messenger RNAs (mRNAs) that are resistant to exonucleases and have higher stability *in vivo*.^1^^,^^2^ Therefore, they are ideal biomarkers and therapeutic targets or tools. circRNAs are widely expressed in mammalian tissues but highly enriched in the central nervous system.^3^ It is involved in various pathophysiological processes by interacting with DNA, RNA, or proteins to regulate transcription and translation.^4^ Recently, the involvement of circRNAs directly translated into peptides or proteins in the pathogenesis of human diseases has attracted extensive attention.^5^, ^6^, ^7^ Research has demonstrated the significance of circRNAs in the pathophysiological mechanisms underlying major depressive disorder (MDD).^8^^,^^9^ However, the function of protein-encoding circRNAs in MDD pathogenesis has not been fully elucidated.

MDD is a common mental disorder that ranks second in the global burden of human disease, affecting approximately 280 million people worldwide.^10^ It is considered a highly heterogeneous disorder triggered by a combination of genetic, environmental, biological, and psychological factors interacting with each other,^11^, ^12^, ^13^ and is usually accompanied by persistent depressive mood and anhedonia. However, its exact pathogenesis is unknown. In the clinic, MDD lacks objective biomarkers for diagnosis, and there are many side effects of drug therapy.^14^^,^^15^ Therefore, it is urgently needed to explore objective biomarkers and effective therapeutic targets.

Our previous study identified a differentially expressed circRNA, circFKBP8(5S,6), which is primate-specific, by whole transcriptome sequencing of peripheral blood from healthy controls and MDD patients.^16^ It was shown to encode a 127-amino-acid protein, cFKBP8, which was increased in both autopsy brain tissue and brain organoids of MDD patients. In addition, circFKBP8(5S,6) was up-regulated in plasma neuron-derived exosomes from MDD patients. Further overexpression of circFKBP8(5S,6) or cFKBP8 in mouse prelimbic cortex (PrL) promoted chronic unpredictable mild stress (CUMS) susceptibility possibly by interfering with glucocorticoid receptor entry.^17^

This study aims to identify the specific molecular mechanisms by which circFKBP8(5S,6) and its encoded protein, cFKBP8, are involved in the pathophysiological processes of MDD using a model animal of depression. Our study demonstrated that circFKBP8(5S,6) and its encoded protein cFKBP8 inhibited neuronal autophagy to promote stress susceptibility in mice by down-regulating the expression of dopamine D3 receptor (DRD3), which in turn modulates the downstream adenosine 5′-monophosphate-activated protein kinase (AMPK)/mammalian target of rapamycin (mTOR)/unc-51-like kinase 1 (ULK1) autophagy signaling pathway.

## Materials and methods

### Animals

Adult male C57BL/6J mice (6–8 weeks old) were purchased from GemPharmatech (Nanjing, China). All mice were housed in a specific pathogen-free animal facility with constant temperature and humidity, a 12 h light/12 h dark cycle, and free access to food and water. Mice were all randomly assigned to groups and underwent a 2-week acclimatization period before the experiment, during which they were handled twice a day. CUMS mice were raised in single cages, and control mice were raised in groups. All mice were deeply anesthetized at the end of the experiment by intraperitoneal injection of 1% sodium pentobarbital solution (70 mg/kg), and then the necks were broken to remove the brains. All experimental procedures followed the regulations of the Institutional Committee for the Care and Use of Animals of Southeastern University School of Medicine (Ethical Permission No. 20200607035).

### CUMS modeling

The CUMS model was established regarding the protocol in the previous study.^18^ Briefly, mice were individually housed and subjected to two randomized mild stressors daily for 5 weeks in the CUMS group and 2 weeks in the sCUMS (short CUMS) group. Five mice were housed per cage and did not receive any stressors in the control group. The stressor was a combination of the following: swimming in warm water at 45 °C for 5 min, swimming in cold water at 4 °C for 5 min, restraint for 2 h, cage shaking for 5 min, tail pinching for 1 min, tail suspension for 6 min, fasting or water fasting for 12 h, moist bedding for 12 h, night lighting for 12 h, tilting the cage for 10 h (at 45°), empty cages for 12 h, stroboscopic lighting for 12 h, and rat bedding for 24 h.

### Sucrose preference test (SPT)

The SPT was used to assess anhedonia in mice. Sucrose solution acclimatization was performed prior to testing, and each mouse was given two bottles of 1% sucrose solution to drink for 48 h. Subsequently, the mice were exposed to one bottle of 1% sucrose solution and one bottle of water for 2 h, and the positions of the two bottles were exchanged for 1 h during the testing. The consumption of sucrose solution and water was measured at the end of the experiment. Sucrose preference was calculated as percentage preference = (sucrose solution intake/total intake) × 100%. The experiment followed a blinded method.

### Forced swim test (FST)

The FST is a reliable method for assessing depression-like behavior in mice. Mice were placed in a clear glass cylinder (height: 25 cm; diameter: 16 cm) with a water level of 15 cm (23°C–25 °C) for 6 min, and only the last 4 min of immobility were recorded. Mice floating motionless on the surface of the water with only their heads exposed and no other activity were considered immobile. Each mouse was wrapped in a towel at the end of the test until its fur was dry and the water was replaced. Experiments were recorded and analyzed using the Any-maze animal behavior analysis system, and followed a blinded method.

### Tail suspension test (TST)

The TST is a widely used test to assess depressive-like behavior in mice. In this experiment, the mice were suspended 50 cm from the ground by tape, which was adhered 2 cm below the tip of the tail for 6 min, and the last 4 min of immobilization were assessed. The experiment was recorded and analyzed using the Any-Maze animal behavioral analysis system, and the blinding rule was followed.

### Stereotactic virus injection

After the mice went through the acclimatization period, they were driven under anesthesia with sevoflurane (Cat: LEG3923, FUJIFILM Wako Pure Chemical Corporation) at a concentration of 1.5 % flow rate of 1 L/min (R580 air anesthesia machine, RWD Life Science), and after testing for good anesthesia, the mice were fixed on a mouse brain stereotaxic apparatus with a digital display (Cat: 68516, RWD Life Science). Body temperature was maintained with heating pads, and eyes were kept moist throughout the surgery. A minimally invasive craniotomy is then performed. Mice were randomly selected and, depending on the grouping, 1 or 2 of the following viruses, vehicle (rAAV-hSyn-bGH-polyA-CMV-EGFP, AAV/9, tilter: 3.00E+12), oe-circ (rAAV-hSyn-circMDD-bGH-polyA-CMV-EGFP, AAV/9, tilter: 2.67E+12), oe-pep (rAAV-hSyn-peptide-3 × Flag-P2A-EGFP-hGH-polyA, AAV/9, tilter: 3.39E+12), and oe-DRD3 (rAAV-hSyn-mDRD3-P2A-mCherry, AAV/9, tilter: 3.35E+12), were injected into bilateral PrL (AP = 1.9 mm; ML = ±0.3 mm; DV = −2.5 mm). The virus was purchased from Brain VTA Technology Co., Ltd. (Wuhan, China). The injection rate was kept at 15 nL/min, and the unilateral injection volume was 300 nL or 600 nL. The injection needle was kept still for 10 min after the injection and then was slowly withdrawn. Table S2 provides the sequence details of the viral vectors.

### Drug administration

To confirm whether activation of DRD3 signaling has a therapeutic effect on the exacerbation of depressive-like behavior in mice induced by circFKBP8(5S,6) or its encoded protein cFKBP8, cariprazine (Cat: HY-14763, MedChemExpress, USA) was dissolved in DMSO and sterile saline, and intraperitoneally injected at a dose of 0.2 mg/kg twice a day starting in the third week of CUMS until the end of modeling. Cariprazine is a partial agonist of dopamine D2 and D3 receptors with high affinity and selectivity for dopamine D3 receptors.^19^ The choice of dose was consistent with previous reports.^20^

### RNA sequencing and analysis

After mice were decapitated, PrL brain tissue was quickly stripped on ice, then thrown into liquid nitrogen for quick freezing, and transferred to a −80 °C refrigerator. RNA was extracted and subjected to quality control. RNA sequencing was performed by Shanghai Genechem Co., Ltd. The raw data were first background calibrated and normalized using the Robust Multi-Array Average (RMA) method with the R package “limma”. Next, the “Hmisc” package was used to identify the missing values. Finally, the differentially expressed genes (DEGs) were acquired using the “limma” package. Genes with |log_2_ fold change| >1 and *P* < 0.05 were regarded as the threshold for DEGs. Volcanic mapping of DEGs was drawn using the R package “ggplot2”.

### Gene Ontology and Kyoto Encyclopedia of Genes and Genomes analysis

The Gene Ontology (GO) is an internationally standardized gene function classification system consisting of biological processes (BP), molecular functions (MF), and cellular components (CC). GO analyzes potential targets of DEGs, and the Kyoto Encyclopedia of Genes and Genomes (KEGG) analyzes their enrichment pathways. Enrichment analysis was performed using the enrichGO and enrichKEGG functions in the R studio package “clusterProfiler”, and the “ggplot2” package was used to visualize the enrichment results. *P*-values < 0.05 were considered statistically significant.

### Total RNA extraction and quantitative reverse transcription PCR

Total RNA extraction and quality determination of PrL tissues were performed according to the manufacturer’s instructions. RNAs of qualified quality were reverse transcribed according to the procedure of HiScript III 1st Strand cDNA Synthesis Kit (+gDNA wiper) (Cat: R312-01, Vazyme). The obtained cDNAs were subjected to real-time fluorescence quantitative PCR using ChamQ SYBR qPCR Master Mix (Cat: Q341-02, Vazyme) on an ABI 7300 Real-Time PCR instrument. The primer sequences used were as follows: DRD3, CCTCTGAGCCAGATAAGCAGC/CCTCTGAGCCAGATAAGCAGC; GAPDH, GGTTGTCTCCTGCGACTTCA/TGGTCCAGGGTTTCTTACTCC. The primers were biosynthesized by GENEWIZ Co., Ltd. The relative expression of genes was calculated using the 2^−ΔΔCT^ method.

### Western blotting assay

The total protein of PrL tissues was lysed and extracted using a RIPA lysis buffer (Cat: P0013B, Beyotime) containing protease and phosphatase inhibitors (Cat: P1048, Beyotime). Protein concentrations were measured using Pierce™ BCA Protein Assay Kits (Cat: 23225, Thermo Fisher Scientific). Proteins were first separated by 4%–20% SDS-PAGE and transferred to a PVDF membrane, which was then blocked with 5% skimmed milk prepared in Tris-buffered saline solution containing 0.1% Tween 20 at room temperature for 1 h. Then, the membranes were incubated at 4 °C overnight with the primary antibodies. The next day, the bands were imaged with the FDBio Dual-ECL Kit (Cat: FD8020, FuDe Biological Technology) and a Tanon 3500 Gel Imaging System after incubating the membranes with goat anti-rabbit secondary antibody or goat anti-mouse secondary antibody at room temperature for 1 h. The grayscale intensities of protein bands were measured using the ImageJ software. Detailed antibody information is provided in Table S1.

### Slice preparation

After the behavioral assessment was completed, the heart was perfused apically with phosphate-buffered saline solution until the effluent was clarified, followed by perfusion fixation in 4% paraformaldehyde (Cat: P0099, Beyotime). Next, the brains were removed and fixed in 4% paraformaldehyde for 2 days before being immersed in 30% sucrose solution. Finally, after embedding the tissue with optimal cutting temperature compound (OCT) (Cat: 4583, SAKURA), 30 μm sections were obtained with a frozen sectioning machine from Thermo Scientific.

### Immunofluorescence

Brain slices were placed in well plates and permeabilized with 0.3% Triton X-100 (Cat: ST795, Beyotime) for 15 min, then blocked with 5% goat serum (Cat: ZLI-9021, Zhongshan Golden Bridge Biotechnology) at room temperature for 1 h, and incubated at 4 °C overnight with primary antibody. The slices were washed repeatedly in a shaker and then incubated with a suitable fluorescent secondary antibody at room temperature for 1 h, and then stained with DAPI (Cat: AB104139, Abcam), and the images were acquired under an Olympus FV3000 confocal microscope. The mean intensity of fluorescence was measured using ImageJ software. Detailed information about the antibodies is provided in Table S1.

### Statistical analysis

GraphPad Prism 9.5 (GraphPad Software, Inc., La Jolla, CA, USA) was used to analyze data, and data were expressed as mean ± standard error of the mean. One-way analysis of variance (ANOVA) followed by Tukey’s post-hoc tests was used for multi-group comparisons. All data are available from at least three independent experiments. *P*-values < 0.05 were considered statistically significant.

## Results

### Identification of potential targets of circFKBP8(5S,6) or its encoded protein cFKBP8 by RNA sequencing

**Figure 1:**
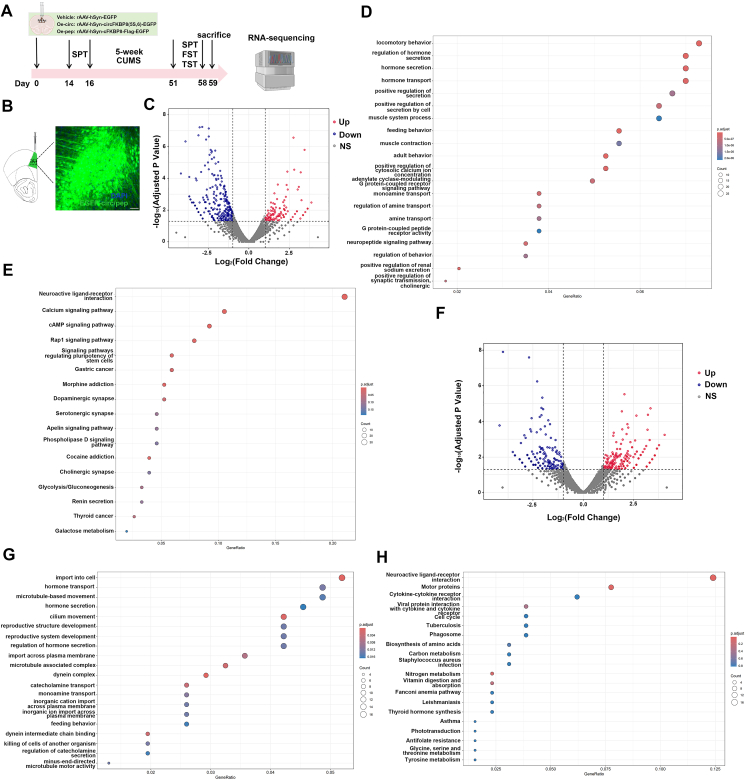
Analysis of DEGs and their functions in PrL after overexpression of circFKBP8(5S,6) or its encoded protein cFKBP8 in CUMS mice. **(A)** Schedule of experimental design and RNA sequencing. **(B)** Representative images showing EGFP expression in the PrL. Scale bar = 50 μm. **(C)** Volcano plot of DEGs after overexpression of circFKBP8(5S,6). **(D)** Bubble diagram of the total analysis of GO enrichment for DEGs after overexpression of circFKBP8(5S,6). **(E)** Bubble chart of KEGG pathway enrichment analysis of DEGs after overexpression of circFKBP8(5S,6). **(F)** Volcano plot of DEGs after overexpression of cFKBP8. **(G)** Bubble graph of overall GO enrichment analysis for DEGs after overexpression of cFKBP8. **(H)** Mapping of the RNA-sequencing data to the KEGG pathway after overexpression of cFKBP8. In the volcano plot, red color intensity signifies up-regulation of gene expression, and blue signifies down-regulation. In the bubble plots of GO enrichment and KEGG enrichment analyses, the size of the dots represents the number of genes, and the color of the dots represents the corrected *P*-value. DEGs, differentially expressed genes; PrL, prelimbic cortex; CUMS, chronic unpredictable mild stress.

In a previous study,^17^ we explored the effects of circFKBP8(5S,6) on depressive-like behavior in mice by injecting recombinant adeno-associated viruses (rAAV) into the PrL of mice to achieve overexpression of circFKBP8(5S,6) or cFKBP8 in neurons. Then, at the end of the 5-week CUMS, tissue was extracted from the PrL brain region for RNA sequencing (Fig. 1A, B). The role of circFKBP8(5S,6) in promoting CUMS susceptibility and exacerbating depressive-like behavior in mice was shown to be dependent on the translational capacity of circFKBP8(5S,6)^17^. These findings have been consistently replicated in our subsequent validation studies. In detail (Fig. S1), the mice overexpressing circFKBP8(5S,6) or cFKBP8 did not show statistically significant differences in behavioral tests such as the SPT, TST, and FST in the absence of CUMS. However, after 5-week CUMS, overexpression of circFKBP8(5S,6) or cFKBP8 induced more severe depressive-like behaviors, including decreased sucrose preference and prolonged immobility time in the TST and FST. We then analyzed the DEGs of the sequencing data for analytical identification. First, we analyzed the circFKBP8(5S,6) overexpression group (CUMS oe-circ group) and the cFKBP8 overexpression group (CUMS oe-pep group) in comparison with the injected control virus group (CUMS vehicle group). As shown in the volcano plot of Figure 1C, our project detected 520 significant DEGs in the CUMS oe-circ group compared with the CUMS vehicle group (|log_2_ fold change| > 1, *P* < 0.05), of which 136 genes were significantly up-regulated and 384 genes were significantly down-regulated (Table S3). Next, we performed GO enrichment analysis and KEGG pathway analysis on these 520 significant DEGs. Figure 1D shows the top 20 overall results from the analysis of GO enrichment, in which locomotory behavior, regulation of hormone secretion, and the G protein-coupled receptor (GPCR) signaling pathway were related to central nervous system function and behavioral regulation. KEGG pathway enrichment analysis revealed that neuroactive ligand–receptor interaction was the pathway most enriched for DEGs (Fig. 1E), which coincidentally corresponded to the GPCR signaling pathway and neuropeptide signaling pathway on the GO enrichment analysis items. Then, we analyzed and compared the CUMS oe-pep group with the CUMS vehicle group (|log_2_ fold change| > 1, *P* < 0.05), and the volcano plot of Figure 1F showed a total of 537 DEGs, including 250 significantly up-regulated genes and 287 significantly down-regulated genes (Table S3). As shown in Figure 1G, 537 DEGs were enriched for import into cells, hormone transport, and hormone secretion by total GO enrichment analysis. Interestingly, KEGG pathway enrichment analysis showed that the DEGs induced by cFKBP8 overexpression were also most significantly enriched in the neuroactive ligand–receptor interaction pathway (Fig. 1H), which is the same as the top 1 pathway analyzed by KEGG pathway enrichment in the circFKBP8(5S,6) overexpression group.

Overexpression of circFKBP8(5S,6) or its encoded protein cFKBP8 down-regulated DRD3 expression and its downstream AMPK/mTOR/ULK1 pathway in PrL of CUMS mice.

**Figure 2:**
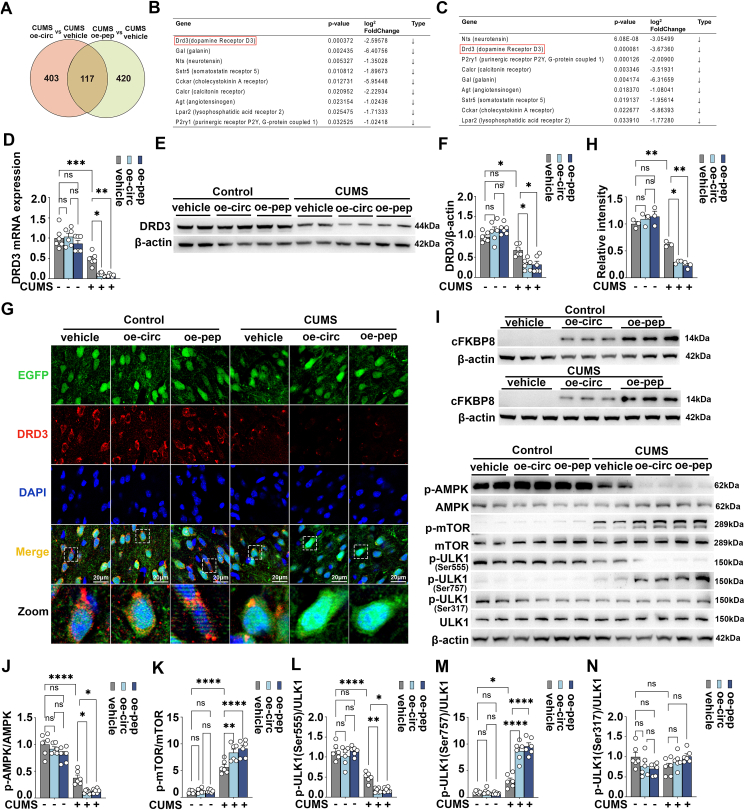
circFKBP8(5S,6) or its encoded protein cFKBP8 down-regulates the expression level of DRD3 and its downstream AMPK/mTOR/ULK1 signaling pathway in PrL of CUMS mice. **(A)** The Venn diagram showed the distribution of the DEGs between circFKBP8(5S,6) and cFKBP8-regulated genes. **(B, C)** DEGs involved in the neuroactive ligand–receptor interaction pathway are co-regulated by cFKBP8 (B) and circFKBP8(5S,6) (C). **(D)** The mRNA levels of DRD3 after overexpression of circFKBP8(5S,6) or overexpression of cFKBP8 in the PrL of mice. *n* = 6. **(E, F)** Western blotting (E) and quantitation (F) for the level of DRD3 in the PrL of mice. *n* = 6. **(G)** Representative immunofluorescence staining images of DRD3 (DRD3-labeling, red) in PrL neurons (EGFP-labeling, green). Scale bar = 20 μm. **(H)** Quantification of the red fluorescence intensity of labeled DRD3 co-labeled with EGFP. *n* = 3. **(I–N)** Representative western blots (I) and quantitative evaluations (J–N) of the cFKBP8, p-AMPK/AMPK, p-mTOR/mTOR, p-ULK1 (Ser555)/ULK1, p-ULK1 (Ser757)/ULK1, and p-ULK1 (Ser317)/ULK1 in the PrL of mice exposed to different treatments. *n* = 6. ∗*P* < 0.05, ∗∗*P* < 0.01, ∗∗∗*P* < 0.001, and ∗∗∗∗*P* < 0.0001; ns, non-significant. Data were represented as mean ± standard error of the mean. DRD3, dopamine D3 receptor; CUMS, chronic unpredictable mild stress; DEGs, differentially expressed genes; PrL, prelimbic cortex; AMPK, adenosine 5′-monophosphate-activated protein kinase; mTOR, mammalian target of rapamycin; ULK1, unc-51-like kinase 1; EGFP, enhanced green fluorescent protein.

Since it has been demonstrated in previous studies that circFKBP8(5S,6) regulation of depressive-like behavior in mice is dependent on its encoded protein cFKBP8,^17^ we took the intersection of DEGs regulated by circFKBP8(5S,6) and cFKBP8. The Venn diagram showed 117 genes that were co-regulated by circFKBP8(5S,6) and cFKBP8 (Fig. 2A and Table S4). We therefore screened DEGs co-regulated by circFKBP8(5S,6) and cFKBP8 from the neuroactive ligand–receptor interaction pathway. DRD3 caught our attention, which ranked first in the cFKBP8 overexpression group and second in the circFKBP8(5S,6) overexpression group and was significantly down-regulated (Fig. 2B, C). Then, we employed western blotting and quantitative reverse transcription PCR to ascertain the DRD3 mRNA and protein expression levels in each group. As shown in Figure 2D–H, overexpression of circFKBP8(5S,6) or cFKBP8 did not affect DRD3 mRNA levels and protein levels in mice without CUMS. In contrast, mRNA and protein levels of DRD3 were significantly decreased in CUMS vehicle mice compared with controls, and interestingly, this change was further exacerbated in CUMS oe-circ mice and CUMS oe-pep mice. The combined DRD3 immunofluorescence results demonstrated that overexpression of circFKBP8(5S,6) or cFKBP8 reduced DRD3 expression in PrL neurons of CUMS mice. We next detected AMPK/mTOR/ULK1 autophagy signaling downstream of DRD3.^21^ Successful translation or overexpression of circFKBP8(5S,6) as well as cFKBP8 was first verified by western blotting (Fig. 2I). As shown in Figure 2J–N, overexpression of circFKBP8(5S,6) or cFKBP8 did not cause changes in the phosphorylation levels of AMPK/mTOR/ULK1 pathway-related proteins in mice without CUMS. Compared with the control group, the levels of p-AMPK and p-ULK1 (Ser555) were significantly decreased and the levels of p-mTOR and p-ULK1 (Ser757) were markedly increased in the CUMS vehicle group, which were further aggravated by overexpression of circFKBP8(5S,6) or cFKBP8. However, the level of p-ULK1 (Ser317) had no significant impact on the groups. Thus, the findings imply that overexpression of circFKBP8(5S,6) or its encoded protein cFKBP8 down-regulated the expression levels of neuronal DRD3 and its downstream AMPK/mTOR/ULK1 (ser757) and AMPK/ULK1 (ser555) pathways in CUMS mice.

### Overexpression of circFKBP8(5S,6) or its encoded protein cFKBP8 down-regulated the level of autophagy in PrL of CUMS mice

**Figure 3:**
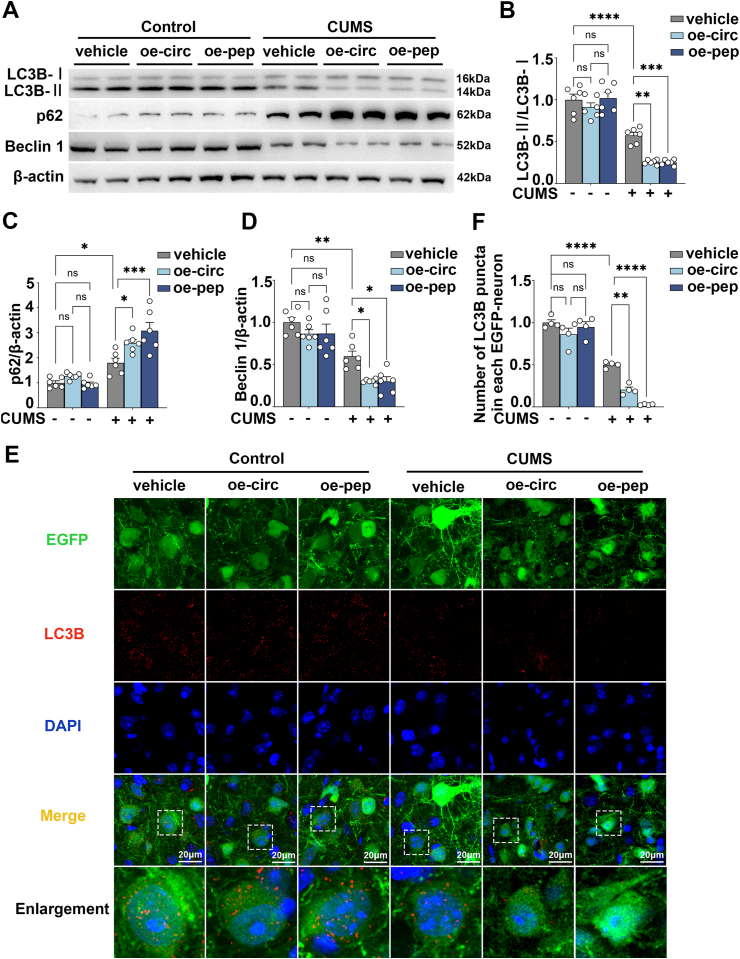
Effects of circFKBP8(5S,6) or its encoded protein cFKBP8 on the expression of LC3B, p62, and beclin 1 in the PrL of mice. **(A**–**D)** Representative western blots (A) and quantitative evaluations (B–D) of the LC3B, p62, and beclin 1 in PrL of CUMS mice exposed to different treatments. *n* = 6. **(E, F)** Representative immunofluorescence staining images (E) and quantitative evaluations (F) of LC3B puncta (LC3B-labeling, red) in PrL neurons (EGFP-labeling, green) in PrL. *n* = 4. Scale bar = 20 μm ∗*P* < 0.05, ∗∗*P* < 0.01, ∗∗∗*P* < 0.001, and ∗∗∗∗*P* < 0.0001; ns, non-significant. Data were represented as mean ± standard error of the mean. LC3B, microtubule-associated protein 1 light chain 3B; PrL, prelimbic cortex; CUMS, chronic unpredictable mild stress; EGFP, enhanced green fluorescent protein.

The AMPK/mTOR/ULK1 pathway is essential for the upstream regulation of autophagy.^22^ Therefore, we assessed the expression levels of the autophagy-related marker genes LC3B (microtubule-associated protein 1 light chain 3B), SQSTM1 (sequestosome 1)/p62, and BECN1 (beclin 1). During the process of autophagy, endogenous LC3B–I tends to be converted to LC3B-II,^21^ therefore, the LC3B-II/LC3B–I ratio was assessed by western blotting (Fig. 3A). The results showed a decrease in the LC3B-II/LC3B–I ratio in the CUMS vehicle group compared with the control group, however, the LC3B-II/LC3B–I ratio in the CUMS oe-circ group and the CUMS oe-pep group decreased more dramatically compared with the CUMS vehicle group (Fig. 3B). SQSTM1/p62 acts as a scaffold between LC3 and ubiquitinated substrates, is incorporated into the autophagosome together, and is degraded by lysosomes along with its ubiquitinated substrates, so that a decrease in SQSTM1/p62 often signals autophagy activation.^23^ Overexpression of circFKBP8(5S,6) or cFKBP8 induced a notable elevation of p62 expression levels in mice undergoing CUMS compared with the CUMS vehicle group (Fig. 3A, C). BECN1 is one of the key regulatory proteins of autophagy and is involved in autophagosome membrane formation.^24^ The expression level of BECN1 was significantly reduced in CUMS vehicle mice, and further reduced in CUMS oe-circ mice and CUMS oe-pep mice (Fig. 3A, D). The number of LC3B puncta reflects the biogenesis and degradation of autophagosomes. As shown in Figure 3E and F, overexpression of circFKBP8(5S,6) or cFKBP8 did not provoke alterations in the number of puncta of LC3B in mice without CUMS. However, CUMS resulted in a decrease in the number of puncta of LC3B, and surprisingly, the decrease in the number of puncta of LC3B was more apparent in the CUMS oe-circ group and the CUMS oe-pep group compared with the CUMS vehicle. This demonstrates that overexpression of circFKBP8(5S,6) or cFKBP8 in PrL neurons of CUMS mice leads to a statistically significant reduction in the number of autophagosomes. Taken together, all these results demonstrate that overexpression of circFKBP8(5S,6) or its encoded protein cFKBP8 inhibits autophagosome synthesis and down-regulates autophagy levels in CUMS mice.

### DRD3 agonist treatment ameliorated the exacerbation of depressive-like behavior induced by overexpression of circFKBP8(5S,6) or its encoded protein cFKBP8, and activated the AMPK/mTOR/ULK1 signaling pathway and neuronal autophagy

**Figure 4:**
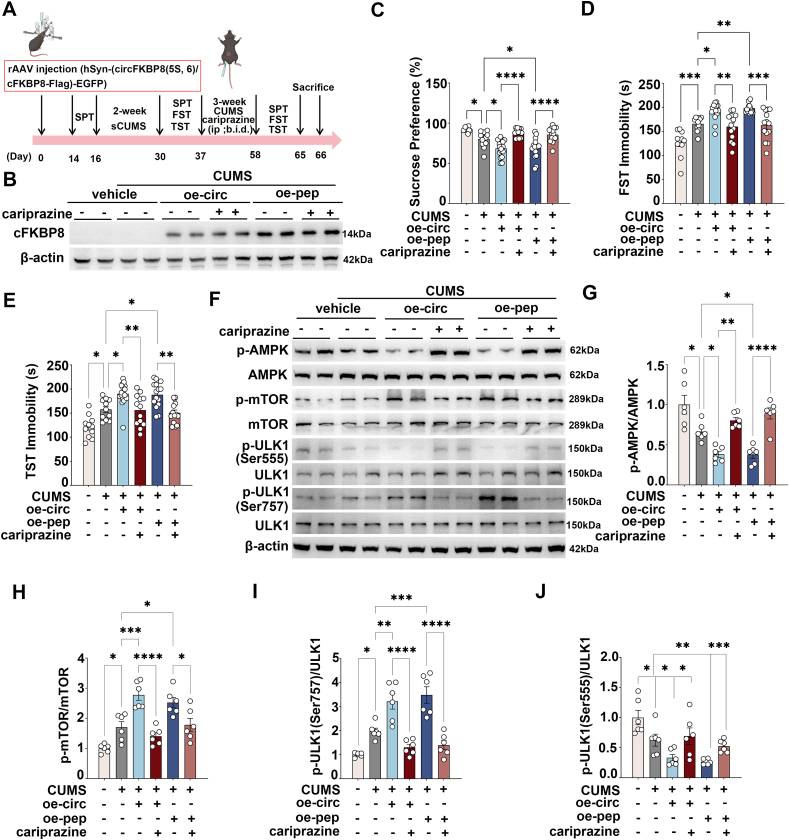
Effects of administration of the DRD3 agonist cariprazine on circFKBP8(5S,6) or its encoded protein-induced depressive-like behavior and the AMPK/mTOR/ULK1 signaling pathway in mice. **(A)** A schematic of the experiment. **(B)** Western blot detection of cFKBP8 expression in each group. **(C)** SPT after intervention with the DRD3 agonist cariprazine. *n* ≥ 11. **(D)** FST after intervention with the DRD3 agonist cariprazine. *n* ≥ 11. **(E)** TST after intervention with the DRD3 agonist cariprazine. *n* ≥ 11. **(F**–**J)** Representative western blots (F) and quantitative analysis (G–J) of the p-AMPK/AMPK, p-mTOR/mTOR, p-ULK1 (Ser555)/ULK1, and p-ULK1 (Ser757)/ULK1 after intervention with the DRD3 agonist cariprazine. *n* = 6. ∗*P* < 0.05, ∗∗*P* < 0.01, ∗∗∗*P* < 0.001, and ∗∗∗∗*P* < 0.0001. Data were represented as means ± standard error of the mean. DRD3, dopamine D3 receptor; PrL, prelimbic cortex; SPT, sucrose preference test; FST, forced swim test; TST, tail suspension test; AMPK, adenosine 5′-monophosphate-activated protein kinase; mTOR, mammalian target of rapamycin; ULK1, unc-51-like kinase 1.

**Figure 5:**
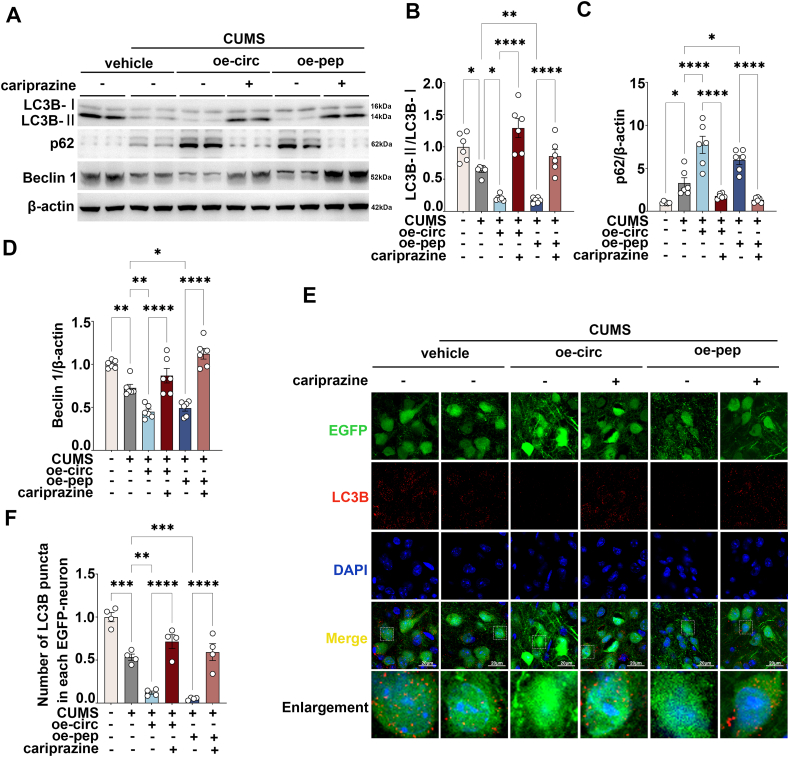
Effects of administration of the DRD3 agonist cariprazine on the level of autophagy induced by circFKBP8(5S,6) or its encoded protein cFKBP8 in mice. **(A**–**D)** Representative western blots (A) and quantitative analysis (B–D) of LC3B, p62, and beclin 1 after intervention with the DRD3 agonist cariprazine. *n* = 6. **(E, F)** Representative immunofluorescence staining images (E) and quantitative evaluations (F) of LC3B puncta (LC3B-labeling, red) in PrL neurons (EGFP-labeling, green) after intervention with the DRD3 agonist cariprazine. *n* = 4. Scale bar = 20 μm ∗*P* < 0.05, ∗∗*P* < 0.01, ∗∗∗*P* < 0.001, and ∗∗∗∗*P* < 0.0001. Data were represented as mean ± standard error of the mean. DRD3, dopamine D3 receptor; LC3B, microtubule-associated protein 1 light chain 3B; EGFP, enhanced green fluorescent protein.

Next, to demonstrate whether circFKBP8(5S,6) or its encoded protein cFKBP8 promotes CUMS susceptibility in mice by down-regulating DRD3 and its mediated AMPK/mTOR/ULK1 signaling pathway and thereby inhibiting autophagy, CUMS mice induced by circFKBP8(5S,6) or its encoded protein cFKBP8 were given intraperitoneal injections with the DRD3 agonist cariprazine (0.2 mg/kg; twice a day) (Fig. 4A). Western blotting was used to verify successful translation or expression of circFKBP8(5S,6) and its encoded protein cFKBP8 (Fig. 4B). Consistent with previous findings,^17^ at the end of 5-week CUMS, more severe depressive-like behaviors were observed in mice neuronally overexpressing circFKBP8(5S,6) or its encoded protein cFKBP8, including decreased sucrose preference and prolonged immobility time in the TST and FST (Fig. 4C–E). However, DRD3 agonist injection rescued this severe depressive-like behavior induced by circFKBP8(5S,6) or its encoded protein cFKBP8 in the SPT, FST, and TST (Fig. 4C–E). Meanwhile, we assessed the phosphorylation levels of AMPK/mTOR/ULK1 pathway-related proteins by western blotting (Fig. 4F). As shown in Figure 4G–J, CUMS treatment resulted in a decrease in the levels of p-AMPK and p-ULK1 (Ser555) and a significant increase in the levels of p-mTOR and p-ULK1 (Ser757) compared with the unstressed group, however, this trend was further exacerbated by the overexpression of circFKBP8(5S,6) or its encoded protein cFKBP8. Interestingly, DRD3 agonists completely reversed the inhibition of the AMPK/ULK1 (Ser555) and the AMPK/mTOR/ULK1 (Ser757) pathways by circFKBP8(5S,6) or its encoded protein cFKBP8 (Fig. 4G–J). More excitingly, DRD3 agonist cariprazine also reversed autophagy levels down-regulated by circFKBP8(5S,6) or its encoded protein cFKBP8. CUMS mice developed autophagy deficits as evidenced by a decrease in the LC3B II/LC3B I ratio (Fig. 5A, B) and LC3B puncta in EGFP-labeled neurons (Fig. 5E, F), an elevated level of p62 expression (Fig. 5A, C), and a decreased level of BECN1 expression (Fig. 5A, D). Overexpression of circFKBP8(5S,6) or its encoded protein cFKBP8 reduced autophagy levels further, however, the DRD3 agonist cariprazine reversed this trend. The above results demonstrated that the DRD3 agonist cariprazine ameliorated severe depression-like behavior induced by circFKBP8(5S,6) or its encoded protein cFKBP8 in mice, and activated the AMPK/mTOR/ULK1 signaling pathway to up-regulate autophagy levels.

### Overexpression of DRD3 alleviated the exacerbation of depressive-like behavior and inhibition of AMPK/mTOR/ULK1 autophagy signaling induced by overexpression of circFKBP8(5S,6) or its encoded protein cFKBP8

**Figure 6:**
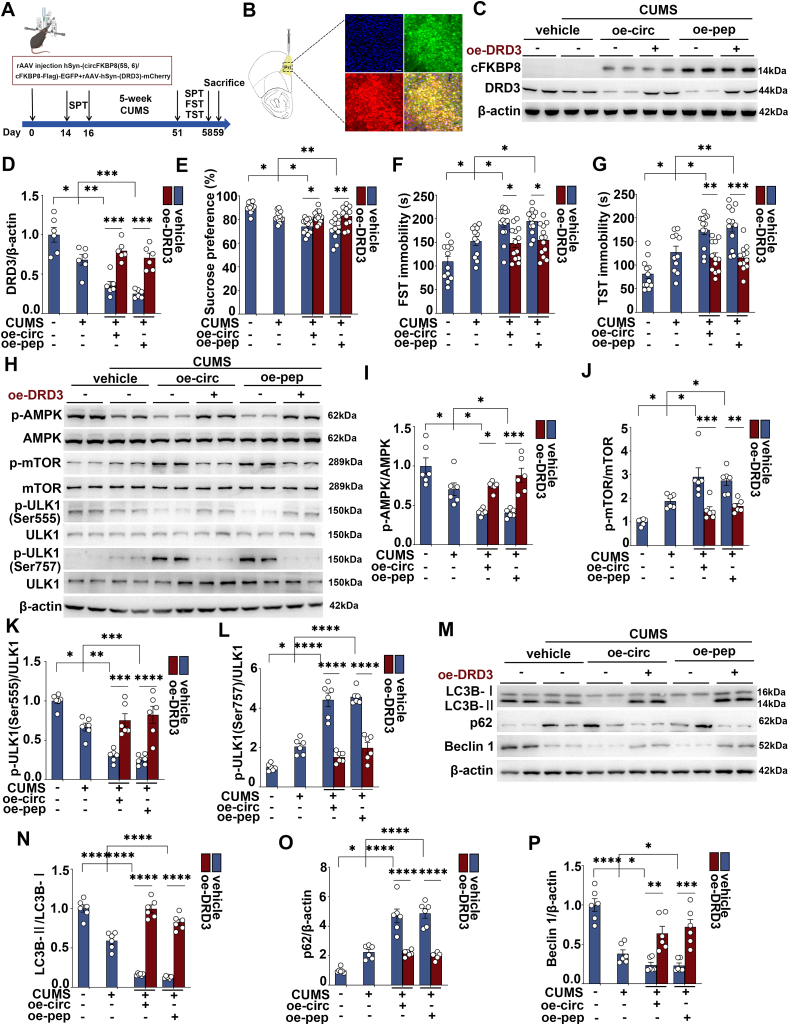
Effects of DRD3 overexpression on depressive-like behavior and AMPK/mTOR/ULK1 autophagy signaling induced by circFKBP8(5S,6) or its encoded protein cFKBP8 in mice. **(A)** A schematic of the experiment. **(B)** Representative images of EGFP and mCherry expression in PrL. Scale bar = 50 μm. **(C)** Western blotting of cFKBP8 and DRD3 expression in each group. **(D)** Quantification of DRD3 by western blotting. *n* = 6. **(E)** SPT after DRD3 overexpression. *n* ≥ 11. **(F)** FST after DRD3 overexpression. *n* ≥ 12. **(G)** TST after DRD3 overexpression. *n* ≥ 11. **(H**–**L)** Representative western blots (H) and quantitative analysis (I–L) of the p-AMPK/AMPK, p-mTOR/mTOR, p-ULK1 (Ser555)/ULK1, and p-ULK1 (Ser757)/ULK1 after DRD3 overexpression. *n* = 6. **(M**–**P)** Representative western blots (M) and quantitative analysis (N–P) of the LC3B, p62, and beclin 1 after DRD3 overexpression. *n* = 6. ∗*P* < 0.05, ∗∗*P* < 0.01, ∗∗∗*P* < 0.001, and ∗∗∗∗*P* < 0.0001. Data were represented as mean ± standard error of the mean. DRD3, dopamine D3 receptor; PrL, prelimbic cortex; SPT, sucrose preference test; FST, forced swim test; TST, tail suspension test; AMPK, adenosine 5′-monophosphate-activated protein kinase; mTOR, mammalian target of rapamycin; ULK1, unc-51-like kinase 1; EGFP, enhanced green fluorescent protein; LC3B, microtubule-associated protein 1 light chain 3B.

We further explored whether DRD3 was involved in the regulation of depressive-like behavior in mice by circFKBP8(5S,6) and its encoded protein cFKBP8. We randomly selected mice to achieve DRD3 overexpression intervention by injecting oe-DRD3 virus (rAAV-hSyn-DRD3-mCherry) at the same time as oe-circ virus (rAAV-hSyn-circFKBP8(5S,6)-EGFP) or oe-pep virus (rAAV-hSyn-cFKBP8-FIag-EGFP) in their bilateral PrL (Fig. 6A, B). The overexpression levels of DRD3 and cFKBP8 were verified by western blotting (Fig. 6C). The expression level of DRD3 was significantly reduced in CUMS mice compared with unstressed mice, and overexpression of circFKBP8(5S,6) or its encoded protein cFKBP8 further reduced the expression level of DRD3 (Fig. 6D). Certainly, as shown in Figure 6D, mice injected with oe-DRD3 virus showed a significant increase in the expression level of DRD3, indicating that DRD3 overexpression was successful. After confirming successful expression of cFKBP8 and DRD3, a series of behavioral assessments including SPT, FST, and TST were performed at the end of the 5-week CUMS. DRD3 overexpression resulted in increased sucrose preference and shortened immobility times in the FST and TST (Fig. 6E–G). Importantly, overexpression of DRD3 reversed the inhibition of the AMPK/mTOR/ULK1 signaling pathway by circFKBP8(5S,6) or its encoded protein cFKBP8, as manifested by the elevation of p-AMPK and p-ULK1 (Ser555) and the decrease of p-mTOR and p-ULK1 (Ser757) (Fig. 6H–L). Moreover, DRD3 overexpression increased the LC3BII/LC3B ratio and BECN1 expression level, decreased the expression level of p62, and reversed the inhibitory effect of circFKBP8(5S,6) or its encoded protein cFKBP8 on autophagy (Fig. 6M−P). Taken together, these strong bases suggest that circFKBP8(5S,6) and its encoded protein cFKBP8 promote stress susceptibility in mice by inhibiting the level of autophagy through down-regulation of DRD3 expression and its downstream AMPK/mTOR/ULK1 signaling pathway.

## Discussion

**Figure 7:**
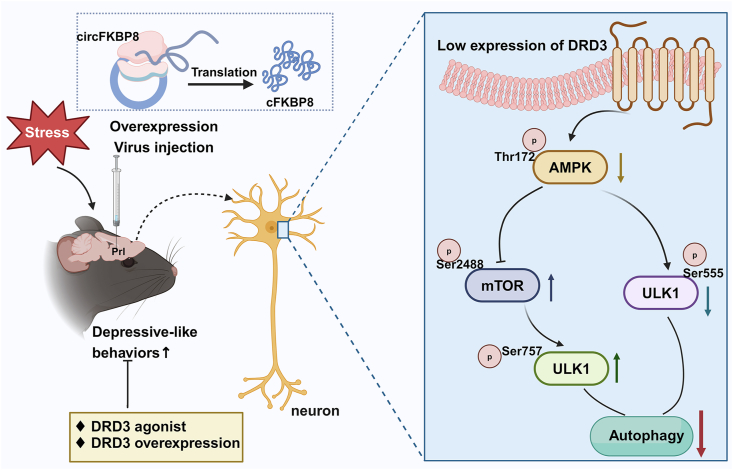
Schematic diagram of the mechanism by which circFKBP8(5S,6) and its encoded protein cFKBP8 promote CUMS susceptibility in mice. circFKBP8(5S,6) or its encoded protein cFKBP8 is known to promote CUMS susceptibility in mice by down-regulating the expression of DRD3 and inhibiting its downstream AMPK/ULK1 (Ser555) pathway and the AMPK/mTOR/ULK1 (Ser757) pathway, which down-regulate neuronal autophagy levels. Figure 7 was created with BioRender.com. CUMS, chronic unpredictable mild stress; DRD3, dopamine D3 receptor; AMPK, adenosine 5′-monophosphate-activated protein kinase; mTOR, mammalian target of rapamycin; ULK1, unc-51-like kinase 1.

The present study elucidated the molecular mechanism by which circFKBP8(5S,6) and its encoded protein cFKBP8 promote CUMS susceptibility in mice. We found that DRD3 expression was significantly reduced in PrL neurons overexpressing circFKBP8(5S,6) or its encoded protein. Further, it was found that the AMPK/mTOR/ULK1 signaling pathway of DRD3 downstream was significantly down-regulated by circFKBP8(5S,6) or its encoded protein cFKBP8, and that the level of neuronal autophagy was also significantly inhibited. Interestingly, both agonists of DRD3 as well as DRD3 overexpression significantly ameliorated severe depressive-like behavior induced by circFKBP8(5S,6) or its encoded protein in mice and up-regulated AMPK/mTOR/ULK1 signaling pathway and activated autophagy. Thus, circFKBP8(5S,6) or its encoded protein promoted CUMS susceptibility in mice by inhibiting neuronal autophagy through down-regulation of DRD3 expression and its downstream AMPK/mTOR/ULK1 pathway (Fig. 7). This study may provide new targets and ideas for the diagnosis and treatment of MDD.

In previous studies, circRNAs are mainly involved in the regulation of transcription and splicing through microRNA sponge adsorption and binding of RNA-binding proteins, thus regulating mRNA stability and translation, interfering with signaling pathways, and participating in the development of diseases.^4^ However, it has recently been gradually recognized that this regulatory mechanism requires circRNAs to adsorb or bind to a large number of microRNAs or RNA-binding protein sites to produce quantitative-level effects.^4^ This has drawn attention to the translational functions of circRNAs. With the development of cutting-edge technologies, studies have successively reported that circRNAs are involved in the regulation of various physiological and pathological processes by encoding peptides or proteins.^25^^,^^26^ Fortunately, our team screened and characterized a circFKBP8(5S,6) that encodes a functional protein of 127aa in a cohort of 7 healthy volunteers and 7 MDD patients.^16^^,^^17^ Upon further investigation, we found that the susceptibility of mice to CUMS was promoted and that loss of translational function abolished this effect. The results of the present study further solidify this conclusion and provide insight into the molecular mechanisms involved.

The DRD3, a member of the D2-like dopamine receptor family, is predominantly distributed in the reward region of the mesolimbic limbic, and this unique distribution determines its potential role in reinforcement and reward.^27^ DRD3 gene polymorphisms are associated with alleles of unipolar major depression, and genetic variants in DRD3 also affect treatment responses to selective serotonin reuptake inhibitors in depressed patients.^28^^,^^29^ In addition, chronic antidepressant medication and chronic electroconvulsive therapy both enhanced DRD3 expression.^30^ Both the expression and function of DRD3 were significantly down-regulated in mice experiencing stress or in a model of depression, which is consistent with our present findings. The difference, however, is that we induced depression-like behavior in mice by CUMS, whereas previous studies were lipopolysaccharide-induced models of inflammatory depression.^31^^,^^32^ circFKBP8(5S,6) and its encoded protein cFKBP8 were the ones that promoted stress susceptibility in mice by further down-regulating the expression of DRD3. Great efforts have been made to search for compounds with high affinity and selectivity to find effective drugs for neuropsychiatric disorders since the distribution and function of DRD3 in these disorders were discovered.^33^ However, little research has been done to address the regulation of DRD3 expression in depression. Our research, however, indicates that circFKBP8(5S,6) or its encoded protein cFKBP8 could down-regulate DRD3 expression in the depressed model mice. This finding offered fresh insights into the molecular regulatory mechanism underlying the aberrant expression of DRD3 in depression as well as novel targets for MDD treatment.

AMPK is a serine/threonine protein kinase consisting of an α catalytic subunit and two regulatory subunits, β and γ. It is known as an energy sensor because of its role in the regulation of energy metabolism.^34^ When the intracellular AMP/ATP ratio is increased, AMPK is activated, as evidenced by the phosphorylation of the threonine 172 residue of the α-subunit.^35^^,^^36^ There is ample research evidence that AMPK plays a key role in the upstream regulation of autophagy.^37^ AMPK, mTOR, and ULK1 are three interrelated nodes that regulate autophagy, of which ULK1 happens to be at the central node of AMPK-regulated autophagy.^22^ AMPK phosphorylates ULK1 in two ways, directly and indirectly: one is mTOR-independent, directly phosphorylating the Ser555 and Ser317 sites of ULK1, and the other is inhibiting the mammalian target of rapamycin complex 1 (mTORC1) by phosphorylating tuberous sclerosis complex 2 (TSC2) and the regulatory associated protein of MTOR complex 1 (Raptor), which in turn reduces the level of phosphorylation of ULK1 at the Ser757 site, and both of these ways can ultimately activate autophagy.^37^^,^^38^ It is worth mentioning that AMPK is also activated in response to activation signals from GPCRs.^36^ It has been reported that DRD3, as a GPCR, activates autophagy through the activation of AMPK and phosphorylation of Raptor, which in turn inhibits mTORC1 and activates ULK1.^39^ In our study, we found that the reduced expression level of DRD3 was accompanied by a repression of the AMPK/mTOR/ULK1 pathway, whereas overexpression of circFKBP8(5S,6) or its encoding protein cFKBP8 further enhanced this change. Several studies have suggested that MDD may be related to the inhibition of the AMPK/mTOR signaling pathway, and some antidepressant drugs and therapies exert their antidepressant effects through the activation of autophagy via the AMPK signaling pathway.^40^, ^41^, ^42^ Autophagy, a lysosomal degradation pathway, is essential for maintaining neuronal homeostasis.^43^^,^^44^ In recent years, many neurological and psychiatric disorders, such as depression, have suggested that activation of autophagy is beneficial for antidepressants.^45^, ^46^, ^47^ In opposition to this, there is research evidence that inhibition of neuronal autophagy is associated with an increase in synaptic proteins.^39^ The neurotransmitter homovanillic acid ameliorated depression-like symptoms in mice by inhibiting synaptic autophagic death and increasing hippocampal synaptic plasticity.^48^^,^^49^ These findings all emphasize the critical role of autophagy in regulating synaptic plasticity and as a key modulator of depression. The LC3B-II/LC3B–I ratio, as well as the reduction of LC3B puncta in neurons, the accumulation of p62, and the reduced expression level of BECN1, all suggest that CUMS-induced neuronal autophagy is impaired and is further exacerbated by circFKBP8(5S,6) or its encoded protein cFKBP8. circFKBP8(5S,6) is expected to be a therapeutic target for depression in the foreseeable future.

There are some limitations of the study. First, the exact molecular mechanism by which circFKBP8(5S,6) and its encoded protein cFKBP8 down-regulate DRD3 is not clear. Interestingly, in prior work,^17^ circFKBP8(5S,6) and its encoded protein were shown to inhibit the entry of glucocorticoid receptors into the nucleus, and nuclear translocation of glucocorticoid receptors regulated the expression of target genes.^50^ Therefore, assuming that DRD3 is a target gene of glucocorticoid receptor, it is likely that circFKBP8(5S,6) and its encoded protein cFKBP8 down-regulate the expression of DRD3 by inhibiting the entry of glucocorticoid receptors into the nucleus. Second, in the DRD3-targeting experiments, the observed behavioral improvements may partially result from the intrinsic antidepressant effects of DRD3 activation or overexpression, as the study design lacked control groups receiving cariprazine treatment alone or DRD3 overexpression alone in the CUMS model. Notably, previous studies have demonstrated that cariprazine’s antidepressant effects in the CUMS paradigm are DRD3-dependent.^20^ Considering that circFKBP8(5S,6) is exclusively expressed in higher primates, the current study employed an rAAV-mediated overexpression system in the mouse PrL to specifically investigate whether circFKBP8(5S,6)’s effects on depression-like behaviors are mediated through DRD3 and its downstream signaling pathways. Third, in the DRD3 agonist experiment, we performed the FST and TST at the midpoint and endpoint of the CUMS paradigm. The acute stress nature of these behavioral tests may complicate the interpretation of the depression-like phenotype, but our comprehensive behavioral assessment strategy, including the SPT, provides a partial mitigation of this limitation.

In conclusion, our findings suggest that circFKBP8(5S,6) and its encoded protein cFKBP8 exacerbated CUMS-induced neuronal autophagy deficits and promoted CUMS-induced susceptibility by down-regulating the expression level of DRD3 and inhibiting its mediated AMPK/mTOR/ULK1 autophagy pathway. This finding strengthens the association of circFKBP8(5S,6) and its encoded protein cFKBP8 with DRD3 and autophagy, and provides an experimental basis for a more comprehensive interpretation and understanding of the involvement of circFKBP8(5S,6) and its encoded protein cFKBP8 in the pathogenesis of MDD, and provides a new way of thinking for finding precise targets for the treatment of MDD.

## CRediT authorship contribution statement

**Dandan Xu:** Writing – original draft, Formal analysis, Data curation. **Zihan Huang:** Data curation. **Gaojia Zhang:** Software, Data curation. **Jiao Jiao:** Data curation. **Yujia Cao:** Formal analysis. **Mengyu Liu:** Data curation. **Yan Kong:** Project administration, Conceptualization. **Zhijun Zhang:** Writing – review & editing, Project administration, Funding acquisition, Conceptualization.

## Data availability

The raw and processed sequencing data from this study have been deposited in the Gene Expression Omnibus (GEO) under accession number GSE294128.

## Funding

This work was supported by the Chinese Science and Technology Innovation 2030—Major Project (No. 2022ZD0211701, 2021ZD0200700), the 10.13039/501100001809National Natural Science Foundation of China (No. 82130042, 81830040, 82371532, 82471552), Shenzhen Science and Technology Serial Funds (Guangdong, China) (No. GJHZ20210705141400002, KCXFZ20211020164543006, JCYJ20220818101615033, ZDSYS20 220606100606014, KQTD20221101093608028), and Jiangsu Province Capability Improvement Project through Science, Technology and Education (China) (No. ZDXK202215).

## Conflict of interests

The authors declared no potential conflict of interests.
