# Supplementary material for: circFKBP8(5S,6)-encoded protein promotes stress susceptibility in mice by down-regulating dopamine D3 receptor expression and its downstream AMPK/mTOR/ULK1 autophagy signaling
Source: Genes Dis. 2025 Jun 18;13(2):101718. doi: 10.1016/j.gendis.2025.101718 (PMC12741372; doi:10.1016/j.gendis.2025.101718)
Supplement: Multimedia component 1 [file mmc1.docx]

***Supplementary Information***

**
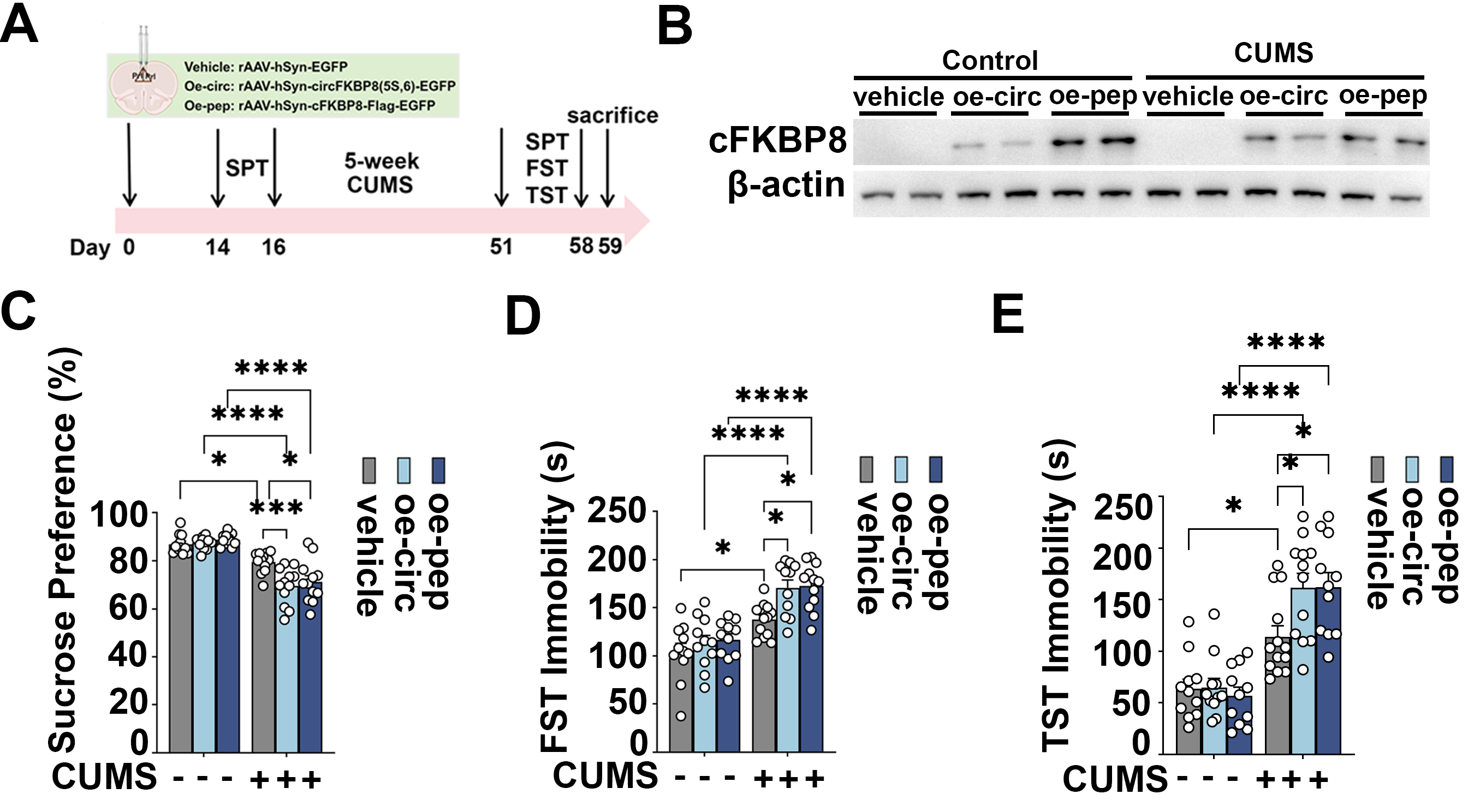
**

**Figure S1. Effects of overexpression of circFKBP8 (5S, 6) or cFKBP8 in PrL brain regions on depressive-like behaviour in mice. (A)** Schedule of experimental design. **(B)** Representative western blot of the cFKBP8, n=6. SPT **(C)**, FST **(D)** and TST **(E)** after CUMS, n ≥ 11, **p* < 0.05, ****p* < 0.001, *****p* < 0.0001. Data are represented as means ± SEM. (PrL, prelimbic cortex; SPT, sucrose preference test; FST, forced swim test; TST, tail suspension test; CUMS, chronic unpredictable mild stress)

**Table S1** Antibodies used in this study.

| Antibodies | Identifier, Source | isotype | Dilution for WB | Dilution for IF |
| --- | --- | --- | --- | --- |
| DRD3 | sc-136170, santa cruz | mouse | 1:500 | 1:100 |
| cFKBP8 | HuaBio | rabbit | 1:200 | - |
| p-AMPK(Thr172) | #4188, Cell Signaling Technology | rabbit | 1:2000 | - |
| AMPK | #5831, Cell Signaling Technology | rabbit | 1:1000 | - |
| p-mTOR(Ser2448) | #5536, Cell Signaling Technology | rabbit | 1:1000 | - |
| mTOR | #2983, Cell Signaling Technology | rabbit | 1:1000 | - |
| p-ULK1(Ser555) | #5869, Cell Signaling Technology | rabbit | 1:1000 | - |
| p-ULK1(Ser757) | #14202, Cell Signaling Technology | rabbit | 1:1000 | - |
| p-ULK1(Ser317) | #89267, Cell Signaling Technology | rabbit | 1:1000 | - |
| ULK1 | #8054, Cell Signaling Technology | rabbit | 1:1000 | - |
| LC3B | NB100-2220, Novus | rabbit | 1:1000 | 1:200 |
| p62 | ab109012, Abcam | rabbit | 1:10000 | - |
| Beclin 1 | ab62557, Abcam | rabbit | 1:1000 | - |
| β-actin | 66009, Proteintech | mouse | 1:5000 | - |
| anti-mouse HRP | SA00001-1, Proteintech | goat | 1:5000 | - |
| anti-rabbit HRP | SA00001-2, Proteintech | goat | 1:5000 | - |
| anti-rabbit alexa-568 | ab175471, Abcam | goat | - | 1:500 |
| anti-mouse alexa-568 | ab175473, Abcam | goat | - | 1:500 |

**Table S2** Sequences for AAV vector.

| Virus | sequence |
| --- | --- |
| oe-circ | GAGCCCATACATCCCCCCGCACGCGGCCCTGTGCCTGGAGGTGACCCTGAAGACGGCTGTGGACGGGCCTGACCTGGAGATGCTCACGGGGCAGGAGCGCGTGGCCCTGGCCAACCGGAAGCGGGAGTGCGGCAACGCCCACTACCAGCGGGCGGACTTCGTCCTGGCCGCCAACTCCTACGACCTCGCCATCAAGGCTATCACCTCCAGCGCCAAAGTGGACATGACGTTCGAGGAGGAGGCACAGCTCCTGCAGTTGAAGGTGAAGTGTCTGAACAACCTGGCGGCCTCGCAGCTGAAGCTCGACCACTACCGCGCAGCCCTGCGCTCCTGCAGCCTTGTGCTGGAGCACCAGCCAGACAACATCAAGGCTCTCTTCCGCAAGGGCAAG |
| oe-pep | ATGCTCACGGGGCAGGAGCGCGTGGCCCTGGCCAACCGGAAGCGGGAGTGCGGCAACGCCCACTACCAGCGGGCGGACTTCGTCCTGGCCGCCAACTCCTACGACCTCGCCATCAAGGCTATCACCTCCAGCGCCAAAGTGGACATGACGTTCGAGGAGGAGGCACAGCTCCTGCAGTTGAAGGTGAAGTGTCTGAACAACCTGGCGGCCTCGCAGCTGAAGCTCGACCACTACCGCGCAGCCCTGCGCTCCTGCAGCCTTGTGCTGGAGCACCAGCCAGACAACATCAAGGCTCTCTTCCGCAAGGGCAAGGAGCCCATACATCCCCCCGCACGCGGCCCTGTGCCTGGAGGTGACCCTGAAGACGGCTGTGGACGGGCCGACTACAAAGACCATGACGGTGATTATAAAGATCATGACATCGATTACAAGGATGACGATGACAAG |
| oe-DRD3 | atggcacctctgagccagataagcagccacatcaactccacctgtggggcagaaaactccactggtgtcaaccgggcccgtccacatgcctactacgccctgtcctactgtgcactcatcctggccatcatctttggcaacggtctggtatgtgcagctgtgctgagggagcgagccctacagaccaccaccaactacctagtggtgagcctggctgtggcagacctgctggtggccactttggtgatgccgtgggtggtgtacttggaggtgacaggtggagtctggaatttcagccgcatttgctgtgatgtttttgtcaccctggatgtcatgatgtgtacagccagcatcctgaacctctgtgccatcagcatagacaggtacacagcagtggtcatgccagttcactatcagcatggcaccgggcagagctcctgtcgacgtgtggcgctcatgattacggctgtgtgggtgctggcctttgctgtgtcctgccctctcctctttggtttcaacacaacaggggatcccagcatctgctccatctccaaccctgattttgtcatttactcttcggtggtgtccttctatgttccctttggggtgactgtcctggtctatgccaggatctacatggtcctgaggcaaaggcgaagaaaacggatcctcactcgacagaacagccagtgtatcagcatcagacctggcttccctcagcagtcttcctgtctgcggctgcatcccattcggcagttttcaataagggccaggtttctgtcagatgccacgggacaaatggagcacatagaagacaaaccatatccccagaaatgccaggaccctctcttgtcacatctacagcccctctctcctggccagacacatggagagctgaaacgctactacagcatctgccaagacactgccttgaggcatccaaacttcgaaggagggggagggatgagccaagtggagaggactcggaactccttaagccccaccatggcacccaagctcagcttagaggttcgaaaactcagcaatggcaggttatccacatccctgaagctggggcccctacagcctcggggagtaccacttcgagagaagaaggccacccagatggtggtcattgtgctcggggccttcattgtctgttggctgcccttcttcttgactcacgttcttaatacccactgtcaggcatgccacgtgtccccagagctttacagagccacgacatggcttggctacgtgaacagtgccctgaaccctgtgatctacaccaccttcaacatagagttccgcaaagccttcctcaagattctatcctgc |
